# Bioengineering the Antimicrobial Activity of Yeast by Recombinant Thanatin Production

**DOI:** 10.3390/antibiotics12121719

**Published:** 2023-12-12

**Authors:** Sofiya O. Pipiya, Arsen M. Kudzhaev, Nisso Z. Mirzoeva, Yuliana A. Mokrushina, Rustam H. Ziganshin, Alexey S. Komlev, Polina E. Petrova, Ivan V. Smirnov, Alexander G. Gabibov, Olga V. Shamova, Stanislav S. Terekhov

**Affiliations:** 1 Shemyakin-Ovchinnikov Institute of Bioorganic Chemistry, Russian Academy of Sciences, Moscow 117997, Russia; kudzhaev_arsen@mail.ru (A.M.K.); mirzoevanis@gmail.com (N.Z.M.); yuliana256@mail.ru (Y.A.M.); ziganshin@mail.ru (R.H.Z.); smirnov@ibch.ru (I.V.S.); gabibov@ibch.ru (A.G.G.); 2 Institute of Experimental Medicine, WCRC “Center for Personalized Medicine”, Saint-Petersburg 197022, Russia; komlev1420@yandex.ru (A.S.K.); polina.petrova@spcpu.ru (P.E.P.); oshamova@yandex.ru (O.V.S.); 3 Department of Chemistry, Lomonosov Mscow State University, Moscow 119991, Russia; 4 Department of Biochemistry, Saint Petersburg State University, Saint-Petersburg 199034, Russia

**Keywords:** antimicrobial peptides, yeast biocontrol agents, recombinant antibiotics, thanatin, antibiotic resistance

## Abstract

The global spread of antibiotic resistance marks the end of the era of conventional antibiotics. Mankind desires new molecular tools to fight pathogenic bacteria. In this regard, the development of new antimicrobials based on antimicrobial peptides (AMPs) is again of particular interest. AMPs have various mechanisms of action on bacterial cells. Moreover, AMPs have been reported to be efficient in preclinical studies, demonstrating a low level of resistance formation. Thanatin is a small, beta-hairpin antimicrobial peptide with a bacterial-specific mode of action, predetermining its low cytotoxicity toward eukaryotic cells. This makes thanatin an exceptional candidate for new antibiotic development. Here, a microorganism was bioengineered to produce an antimicrobial agent, providing novel opportunities in antibiotic research through the directed creation of biocontrol agents. The constitutive heterologous production of recombinant thanatin (rThan) in the yeast *Pichia pastoris* endows the latter with antibacterial properties. Optimized expression and purification conditions enable a high production level, yielding up to 20 mg/L of rThan from the culture medium. rThan shows a wide spectrum of activity against pathogenic bacteria, similarly to its chemically synthesized analogue. The designed approach provides new avenues for AMP engineering and creating live biocontrol agents to fight antibiotic resistance.

## 1. Introduction

The discovery of antibiotics marked a milestone in the victory of medicine over pathogens. This has saved millions of lives and improved the quality of life for even more people. However, due to the spread of multidrug-resistant bacteria, conventional antibiotic therapy is becoming increasingly ineffective [1]. The overuse of antibiotics, as well as the decreasing number of antimicrobials approved for clinical use, only contributes to the further distribution of MDR pathogens. This problem stimulates the search for alternative sources of antimicrobial compounds [2].

Antimicrobial peptides (AMPs) represent a highly diverse class of DNA-encoded antimicrobials that still have great potential as drug candidates. The rapid bactericidal mechanism of action allows AMPs to effectively combat bacteria, including biofilm eradication [3]. Generally, AMPs have alternative mechanisms of action compared to conventional antibiotics [4]. Most conventional antibiotics act on bacterial ribosomes [5,6], transpeptidases [7], and topoisomerases [8]. AMPs use alternative molecular targets, contributing to a lower level of bacterial resistance, acting by direct killing by membrane permeabilization [9,10,11] or inhibiting the activity of essential molecular targets in bacterial cells [12] like Lpt proteins [13]. Resistance to conventional antibiotics has been widely confirmed in clinical settings, whereas data regarding antimicrobial peptides are limited. This could be due to the low level of AMP application in clinical practice [14]. Nonetheless, laboratory studies indicate a lower rate of developing resistance to AMP with a relatively low increase in MIC fold [15]. Thus, AMPs have the potential to limit the spread of antibiotic resistance. AMPs are well known for their good activity against multidrug-resistant (MDR) bacteria [16], as well as for their ability to restore the sensitivity of pathogens to antibiotics [17]. A synergistic effect of AMPs with polymyxin B, tetracycline, and erythromycin was shown on the MDR *Pseudomonas aeruginosa* isolate (PA910) [18]. The broad activity spectrum, killing-based activity mechanism, and high potential against MDR bacteria make AMPs promising drug candidates.

Thanatin is a short beta-hairpin peptide (21 amino acid residues) isolated from the spined soldier bug (*Podisus maculiventris*) [19]. Thanatin is active against both Gram-negative and Gram-positive bacteria, including MDR clinical isolates of *Enterobacter aerogenes* and *Klebsiella pneumoniae* [20]. Several mechanisms of action of thanatin on a bacterial cell were proposed to explain such a wide range of sensitive bacteria. Thanatin displaces divalent metal ions from the bacterial outer membrane, leading to membrane destabilization [21]. It also substitutes zinc ions from the active site of New Delhi metallo-β-lactamase-1 (NDM-1) and restores the sensitivity of the bacterial strain to beta-lactam antibiotics [21]. Thanatin disrupts the operation of the lipopolysaccharide (LPS) transport machinery by interfering with interactions between Lpt proteins, resulting in bacterial cell death [22]. The multiple actions of thanatin on bacteria make it a promising object for the creation of novel antimicrobials. Thanatin is active in a broad pH range and it is inhibited at a high salt concentration [23].

One of the main factors limiting the widespread use of AMPs is the high cost of their production [24]. Peptides with sophisticated structures require complex chemical synthesis strategies, which are time-consuming and expensive [25]. To overcome this limitation, it is necessary to develop cost-effective ways to produce AMP. The heterologous production of recombinant AMPs is widely used as an alternative to chemical synthesis. One of the most common hosts for heterologous protein production is *Escherichia coli*, which allows fast and inexpensive recombinant protein production [26]. However, AMP production in *E. coli* is often limited due to the self-toxicity of recombinant AMPs. Fusions with other proteins can overcome this limitation, but in this case, additional purification steps are required [27]. Several AMPs have been successfully produced in yeasts, such as apidaecin [28], mytichitin-A [29], shakin-1 [30], and the fusion protein cecropinA-thanatin [31], supporting the importance of the biotechnological production of antimicrobials. The overwhelming majority of studies pay primary attention to the biotechnological production of AMPs using methanol-inducible AOX1 promoter systems. The inducible AOX1 promoter provides high yields, especially in the case of toxic polypeptides. However, the addition of toxic methanol to the growth medium is needed. Hence, these recombinant AMP producers could not be considered biosafe biocontrol agents, and they do not provide a self-propagating source of AMPs.

Here, we propose an alternative source of recombinant thanatin (rThan) based on constitutive production in the yeast *Pichia pastoris*. Yeasts as host organisms are a convenient choice for their rapid growth and high yields of recombinant proteins [32], and they are expected to be less vulnerable to AMP toxicity [33]. rThan was obtained with a high overall yield, reaching up to 20 mg of the purified peptide per liter of growth culture. The resulting peptide had broad antimicrobial activity against a panel of bacterial pathogens, similarly to the chemically synthesized analog. The genetically encoded nature of AMPs enables the construction of live biocontrol agents for bacterial killing [34]. Recombinant yeast producing rThan efficiently inhibited the growth of bacteria and eradicated bacteria in coculture. The engineering of live biocontrol agents by heterologous AMP production will simplify their production and development, stimulating further achievements in the field of synthetic biology of AMPs.

## 2. Results

### 2.1. Bioengineering rThan—Producing Yeast

Utilizing bioengineering techniques to give organisms novel characteristics creates new opportunities for the application of recombinant organisms. This study is dedicated to the development of a continuous, self-renewing source of antimicrobial activity that does not require the addition of an inducer. Yeast *P. pastoris* is a good candidate for creating such a bioengineering pipeline because of its wide range of genetic engineering toolkits and simplicity of use.

**Figure 1:**
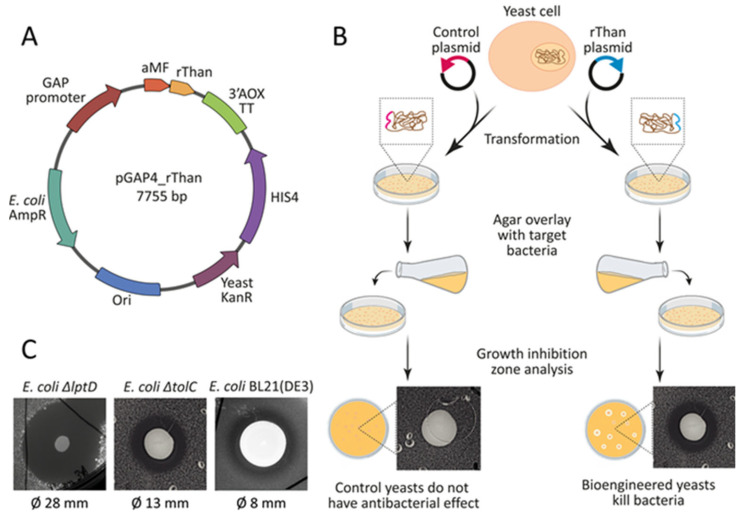
(**A**) A schematic representation of the rThan expression vector. P_GAP_—GAP promoter, aMF—secretion signal sequence, rThan—thanatin coding sequence, 3′AOX TT—AOX1 transcriptional terminator, HIS4—histidinol dehydrogenase, KanR—yeast kanamycin resistance, AmpR—*E. coli* ampicillin resistance, Ori—*E. coli* origin of replication. (**B**) A schematic representation of the yeast bioengineering workflow. Yeast cells were transformed with the target plasmid. The resulting colonies were overlaid with bacteria-inoculated agar. Clones with clear growth inhibition zones were used for further investigations. The control plasmid containing fluorescent protein mCherry was described previously [35]. Agar overlay was performed with *E. coli* Δ*tolC* as the target bacteria. (**C**) Antimicrobial activity of rThan-producing yeasts, estimated by agar overlay assay using target strains *E. coli* Δ*lptD, E. coli* Δ*tolC,* and *E. coli* BL21(DE3). The rThan-producing yeasts formed transparent zones of inhibition.

The yeast expression vector pGAP4_rThan was constructed to provide a strong constitutive expression of the rThan transgene in the yeast *P. pastoris* (Figure 1A). The genetic sequence of thanatin was codon-optimized and cloned under the control of a strong constitutive glyceraldehyde-3-phosphate dehydrogenase (GAP) promoter. The yeast alpha-mating factor signal sequence (aMF) provided the secretion of mature rThan into culture media. Constitutive AMP production enables *P. pastoris* to be provided with antibacterial properties that were estimated by an agar overlay assay (Figure 1B). rThan-producing yeasts inhibited the growth of *E. coli* strains, including hypersensitive *E. coli* Δ*lptD*, *E. coli* Δ*tolC*, and wild-type *E. coli* BL21(DE3)*,* giving transparent clearance zones with a diameter of 28 ± 2, 13 ± 1, and 8 ± 1 mm, respectively (Figure 1C). Clones with the largest zone of growth inhibition were used for further production and purification of rThan.

### 2.2. Constitutive Production and Purification of rThan

**Figure 2:**
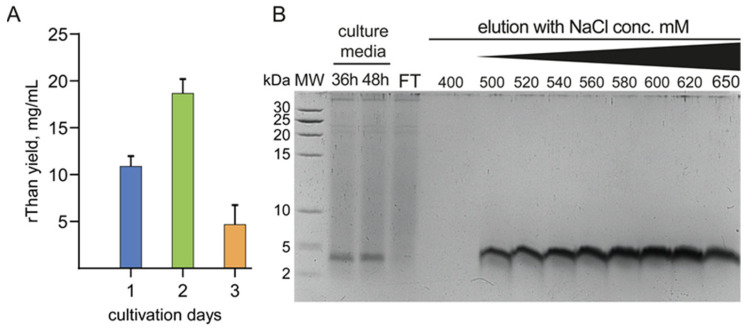
Production and purification analysis of rThan: (**A**)—Time-dependent rThan production analysis, where columns represent rThan concentrations in culture media at different time points; (**B**)—Tricine-SDS-PAGE analysis of rThan purification: MW—Protein MW marker; FT—SP-sepharose flowthrough; 400—concentration of NaCl in elution buffer(mM); 500–650—part of a linear gradient of an elution buffer with increasing NaCl concentration from 500 to 650 mM. Data represent the mean of three biological replicates ± SD.

Constitutive expression of AMP has minimum and maximum production yields upon cultivation time. Different timepoints were checked to determine the production peak. The highest rThan concentration was obtained on the second day of cultivation (Figure 2A) and these probes were subjected to purification.

Cation exchange chromatography was applied for rThan extraction and purification since it has an expected positive charge of +6 at pH 6.0. Strong binding of rThan to cation exchange resin provided its elution at high salt concentrations (from 500 to 650 mM NaCl), resulting in highly purified peptide fractions (Figure 2B). The overall yield of rThan was estimated as 20 mg/L after the purification steps. The proposed production and purification scheme provides a cost-effective source of rThan with a minimum number of purification stages, which is also economically advantageous.

The concentration of rThan in culture media decreased after three days of cultivation, which may be mediated by the proteolytic degradation of peptide by secreted yeast proteases. Nevertheless, after purification steps and storage at the described conditions, rThan was stable and did not show any change in concentration or activity for months.

### 2.3. Chemical Synthesis of Thanatin

**Figure 3:**
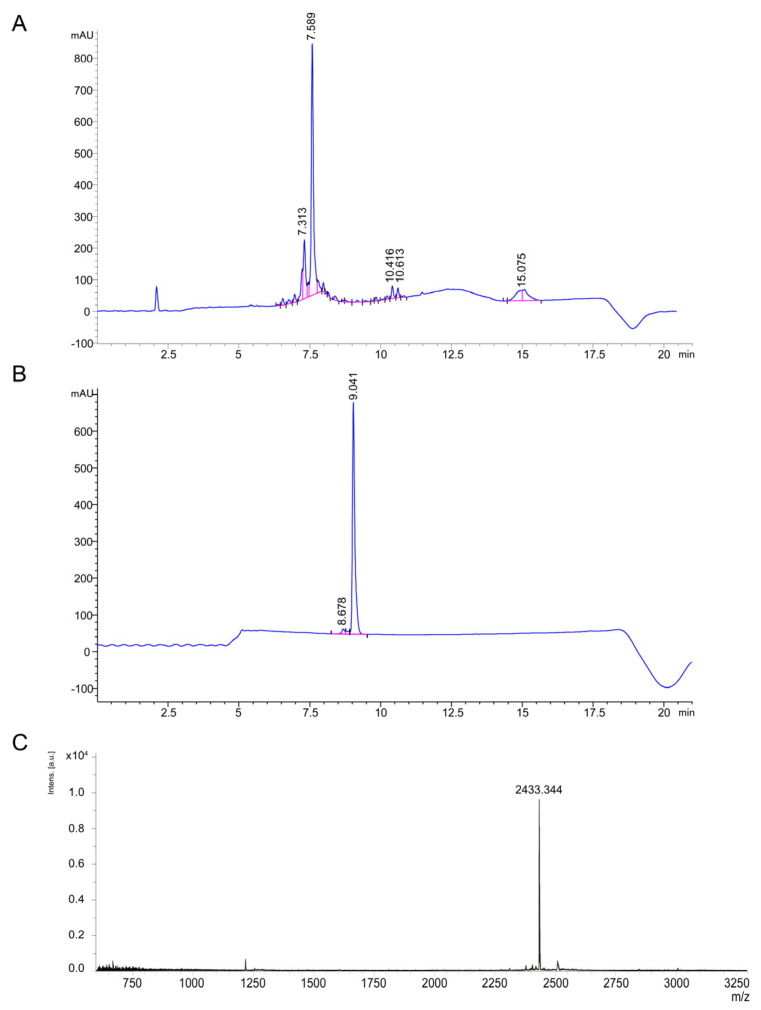
Chemical synthesis of thanatin. (**A**) HPLC profile of crude linear thanatin (53% purity): C18 column Phenomenex Luna (130 Å, 3.5 μm, 4.6 × 250 mm); temperature: 35 °C; flow: 1.0 mL/min; eluent: 0.1% (*v*/*v*) TFA in H_2_O (buffer A) and 0.1% (*v*/*v*) TFA in CH_3_CN (buffer B), λ 220 nm; gradient: 5−60% of buffer B in 20 min. (**B**) HPLC profile of purified cyclized thanatin (95.7% purity): C18 column Phenomenex Luna (130 Å, 3.5 μm, 4.6 × 250 mm); temperature: 35 °C; flow: 1.0 mL/min; eluent: 0.1% (*v*/*v*) TFA in H_2_O (buffer A) and 0.1% (*v*/*v*) TFA in CH_3_CN (buffer B), λ 220 nm; gradient: 5−60% buffer B in 20 min. (**C**) MALDI-TOF mass spectrum of cyclized sThan: *m*/*z* 2433.34—experimental data for [M + H]^+^, 2433.28—theoretical.

To compare rThan with synthetic thanatin (sThan), the latter was obtained by chemical synthesis. Crude thanatin was synthesized by standard solid-phase peptide synthesis, resulting in a linear sThan precursor (Figure 3A).

Cyclized sThan was obtained by the oxidation of a linear sThan precursor, with a 24% overall yield (Figure 3B). The obtained molecular weight of sThan *m*/*z* _[M + H]_^+^ = 2433.34 was in line with its theoretical mass *m*/*z* [M + H]^+^ = 2433.28 (Figure 3C).

LC-MS analysis of purified rThan confirmed the same *m*/*z* [M + H]^+^ = 2433.30 Da, indicating its uniformity with sThan (Figure S1). Hence, the developed strategy of constitutive production of antimicrobial peptide results in active full-length rThan that could be easily isolated from culture media with high purity.

### 2.4. Antimicrobial Activity Spectra of rThan

**Figure 4:**
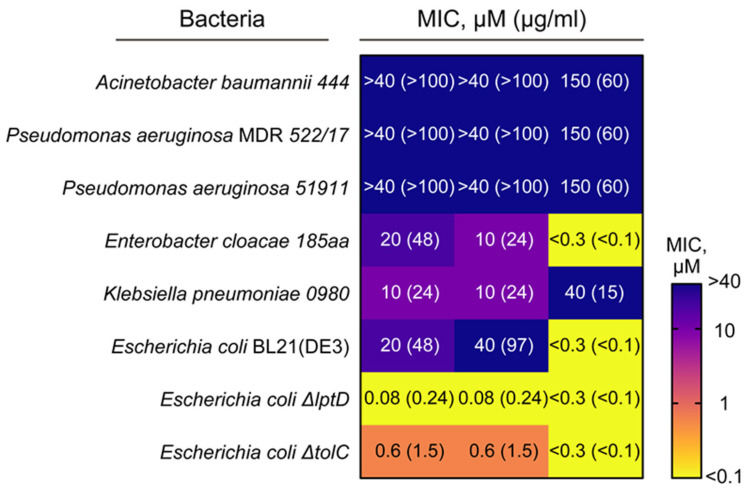
Minimal inhibitory concentrations (MICs) of rThan and sThan toward a panel of Gram-negative pathogens and model *Escherichia coli* strains.

Antimicrobial activity testing was performed with a panel of pathogenic bacteria (including MDR strains) and model *E. coli* strains to estimate the antimicrobial activity of sThan and rThan produced by *P. pastoris* (Figure 4). The conventional antibiotic ciprofloxacin was used as a control.

Recombinant rThan displayed similar activity to chemically synthesized sThan. The identified MIC values showed that thanatin has potent activity against bacteria from the *Enterobacteriaceae* family. Both sThan and rThan were more active than ciprofloxacin against the multidrug-resistant (MDR) strain *K. pneumoniae* 0980 (*p* = 0.028, Mann–Whitney test), demonstrating median MIC values of 10 µM, 10 µM, and 40 µM, respectively. Thanatin and ciprofloxacin have different mechanisms of action [21,22,36], which mediate reasonable rThan activity toward bacteria resistant to fluoroquinolone antibiotics. Therefore, thanatin provides a new therapeutic option as an alternative to conventional antibiotics. Thanatin was not active against MDR stains *A. baumannii* 444, *P. aeruginosa* 51911, and *P. aeruginosa* 522/17 under the tested conditions. Both sThan and rThan were inactive against the Gram-positive bacteria *Staphylococcus haemolyticus* 515, *Bacillus cereus* X1, *Enterococcus faecalis* 125, and *Staphylococcus aureus* GFP at concentrations up to 40 µM, confirming its LPS-mediated mechanism of action.

Model *E. coli* strains were used to discriminate between permeability and transport effects on thanatin activity. *E. coli* Δ*lptD* strain has increased permeability of the outer membrane, and *E. coli* Δ*tolC* lacks efflux machinery, generally resulting in increased sensitivity to antibiotics. One of the proposed mechanisms of thanatin action is based on dysregulation of the LPS transport system (Lpt) [22]. Hence, thanatin was particularly active against *E. coli* Δ*lptD*, resulting in a dramatic decrease in MIC compared to *E. coli* BL21(DE3). Abolishing the antibiotic efflux machinery in *E. coli* Δ*tolC* has a lesser impact on thanatin susceptibility, indicating that membrane permeability plays the main role in the activity of thanatin against Gram-negative pathogens. Thus, AMPs having an increased permeability toward the outer membrane and targeting the LPS transport system (Lpt) have a high potential to become effective drug candidates.

No effect on the viability of the human cell line HEK293T was detected at concentrations of rThan and sThan up to 256 µM.

### 2.5. Cocultivation Experiments Reveal Biocontrol Potential of rThan-Producing Yeasts

Constitutive production of rThan allows the detection of antimicrobial activity directly from culture media. Hence, rThan-producing strains act as bioengineered biocontrol agents that inhibit target bacteria in coculture.

**Figure 5:**
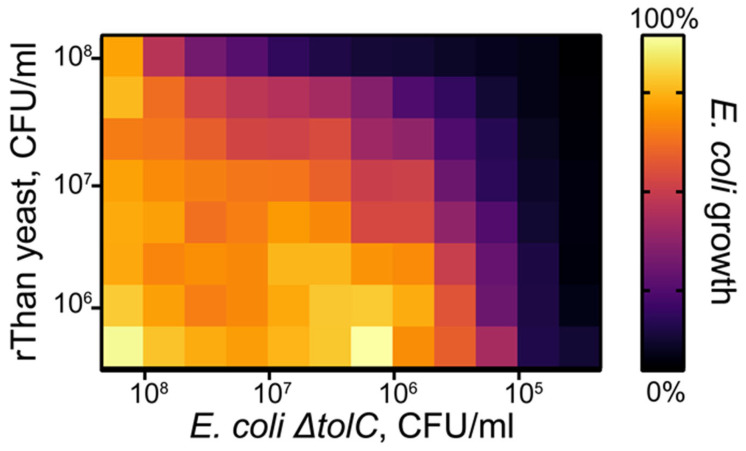
*E. coli* Δ*tolC* sfGFP growth inhibition landscape. rThan-producing *P. pastoris* was cocultivated with GFP-producing *E. coli* Δ*tolC* at different starting cell concentrations. Growth inhibition of target bacteria was estimated according to the level of the GFP fluorescence signal: from complete growth inhibition (black color) to no inhibition (light yellow color). CFU/mL data indicate initial cell concentrations. The detection limit of viable *E. coli* Δ*tolC* sfGFP cells is approximately 10^3^ CFU/mL.

To estimate the inhibitory landscape of rThan-producing yeast, a cocultivation assay was performed with different yeast:bacteria ratios. *E. coli* Δ*tolC* strain expressing sfGFP was used as a live biosensor bacterium and survival was estimated according to the fluorescence signal (Figure 5).

rThan-producing *P. pastoris* inhibited reporter *E. coli* Δ*tolC* at various cell concentrations after overnight incubation. The most pronounced inhibitory effect was observed at a yeast concentration of 10^8^ CFU/mL, which provided inhibition of bacteria at titers up to 10^7^ CFU/mL. At lower yeast titers, a clear inhibition of bacterial growth was observed in the range of 3·10^5^ to 10^6^ CFU/mL. Thus, recombinant yeasts were shown to effectively inhibit the growth of the tested model bacteria. This approach provides a convenient model for the design of probiotics based on recombinant AMP-producing microorganisms. However, further optimization of cultivation and probiotic characteristics is required.

## 3. Discussion

Antibiotic resistance is spreading among pathogens and poses a serious threat to public health. In 2015, the World Health Organization (WHO) declared the fight against antibiotic-resistant pathogens a priority global problem. WHO released a list of priority pathogens against which it is necessary to develop antimicrobials in the first place [37]. So-called ESCAPE pathogens include *Enterococcus faecium*, *Staphylococcus aureus*, *Klebsiella pneumoniae*, *Acinetobacter baumannii*, *Pseudomonas aeruginosa*, and Enterobacter species. Many strains of these bacteria also exhibit multidrug resistance. At the same time, the mechanisms of resistance to antibiotics are highly diverse: these include direct modification of the antibiotic, changes in the structure of the target binding site with the antibiotic, and a decrease in the influx of the antibiotic into the cell due to changes in the operation of transporters, as well as the formation of biofilms [38]. Thus, new antimicrobials must have alternative mechanisms of action in order to overcome bacterial resistance. Antimicrobial peptides have alternative mechanisms of action compared to conventional antibiotics and therefore are ideal candidates for new antimicrobial drugs.

Clinical trials require a large amount of the test substance, so the development of methods for obtaining antibiotics in large quantities is an urgent task. This work focused on the production of the antimicrobial peptide thanatin in the cells of the methylotrophic yeast *Pichia pastoris*. We have shown that the constitutive production of the peptide under the control of the GAP promoter in the framework of fed-batch cultivation makes it possible to obtain up to 20 mg/L of the purified peptide. Yeast cell factories for the production of recombinant proteins are a good alternative to *E. coli* expression systems and mammalian cells. They can rapidly increase biomass and, unlike mammalian cell lines, do not require expensive media and equipment. At the same time, the native disulfide bond formation allows them to obtain a rThan similar to chemically synthesized sThan that does not require refolding, as was reported for *E. coli* cells [39]. The human cell line HEK293 was used for thanatin production [40]. However, mammalian cell culturing elevates production costs dramatically. Moreover, it could not be applied to the creation of biocontrol agents. Examples of the production of antimicrobial peptides in yeast are known, but for some AMPs, the yield is approximately hundreds of micrograms per liter, as in the case of protegrin [41] or melittin [33]. We assume that the high production of thanatin in the *P. pastoris* system is associated with its reduced toxicity towards yeast cells. Studies of thanatin show that it has a low level of hemolysis [42] and low toxicity to mammalian cells [43]. This reduced toxicity may be because the main target of thanatin is the Lpt system, which is absent in eukaryotes.

*K. pneumoniae* and *Enterobacter* spp. are dangerous pathogens, among which multiresistant strains are often found. In this work, it was shown that the obtained purified rThan can inhibit the growth of *K. pneumoniae* and *E. cloacae* with MIC values of 10 and 20 μM, respectively, which are similar to those for sThan. Thus, the yeast system can be used for the effective production of thanatin, and the resulting peptide exhibits antimicrobial activity similar to that of the chemically synthesized analog.

The beneficial feature of antimicrobial peptides is their genetically encoded nature, which allows the use of a vast arsenal of molecular biology tools to create genetically modified producers that efficiently counteract pathogenic microorganisms. Previously, yeast *P. pastoris* was modified for biocontrol of the phytopathogen mold *Penicillium expansum* [44], Gram-positive pathogenic bacteria *Staphylococcus aureus* [35], and Gram-negative *E. coli* strains [34]. The biocontrol agents described above are applied to target specific pathogens and, as in the case of antipenicillium yeasts, they require inductor addition. The low cytotoxicity toward mammalian cells and exceptional specificity of thanatin toward bacterial membranes [22] provide beneficial features for the development of thanatin-based biocontrol agents. Although the probiotic features of *P. pastoris* were not extensively studied, there are several reports speculating their probable application as probiotics [45] and highlighting their safety [46]. Here, we combined the high potential of yeast bioengineering with the exceptional antimicrobial activity of thanatin, associated with low toxicity, to broaden the arsenal of designer biocontrol agents and provide new AMP applications. Hence, the further development of living biocontrol agents is of particular interest. Yeast bioengineering provides a simple and efficient tool for their design. Here, we demonstrated that the cocultivation of thanatin-producing yeast with model strain *E. coli* Δ*tolC* leads to inhibition of the growth of the target bacterium. This effect is observed at various concentrations of the effector and target, providing a highly promising concept of designer living agents for the biocontrol of pathogens. Further studies on the effects of the combination of bioengineered microorganisms with other antimicrobial agents will propose novel therapeutic strategies for the treatment of infectious diseases.

## 4. Materials and Methods

### 4.1. Bacterial and Yeast Strains

A bacterial strain collection including clinical isolates of *Pseudomonas aeruginosa 51911*, *Klebsiella pneumoniae 0980*, *Acinetobacter baumanii 444*, *Enterobacter cloaceae 185aa*, *Staphylococcus haemolyticus 515*, *Bacillus cereus X1*, and *Enterococcus faecalis 125* was kindly provided by Lytech Co. Ltd. (Moscow, Russia). *Escherichia coli* Δ*lptD* and *Escherichia coli* Δ*tolC* were kindly provided by Professor Osterman I.A. and *Pseudomonas aeruginosa* MDR 522/17 was kindly provided by Professor Shamova O.V. *Staphylococcus aureus* GFP was described previously [35]. *Escherichia coli* BL21(DE3) (Evrogen, Moscow, Russia) and *Escherichia coli* XL-Blue (Evrogen, Russia) were used for plasmid cloning. *Pichia pastoris* GS115 was used as a host organism for secreted rThan production.

### 4.2. Expression Vector Design and Construction

The expression vector was constructed from two parts.

For the first part, the intermediate vector pLvL1_GAP_MCS was prepared. Assembly plasmids (Supplementary Table S1) from MoClo yeast [47] and MoClo pichia toolkits [48] were used to generate the intermediate plasmid pLvL1_GAP_1 according to the protocol [47]. The resulting plasmid was amplified with PCR primers P1 and P2. The multiple cloning site (MCS) sequence was synthesized by PCR using Primerize-predicted primers P3 and P4 [49]. Both fragments were purified with the Cleanup kit (Evrogen, Russia) and subjected to the HIFI DNA assembly reaction (New England Biolabs, Hitchin, UK) followed by the transformation of *E. coli* XL-Blue. The resulting vector pLvL1_GAP_MCS was verified by sequencing.

For the second part, a region of the pPIC9K plasmid, including TT (transcription terminator) and HIS4 sequences, was amplified by overlapping PCR to destroy the BsaI restriction site at the HIS4 gene with primers P5–P8. The obtained PCR fragment (Fr1) was purified with the Cleanup kit (Evrogen, Russia) for further cloning.

The pGAP4_MCS plasmid was constructed based on pLvL1_GAP_MCS and Fr1. The fragment of pLvL1_GAP_MCS was PCR amplified with primers P9 and P10 and ligated with Fr1 using the HIFI DNA assembly reaction (New England Biolabs, UK). The resulting expression vector pGAP4_MCS was identified by PCR and verified by sequencing.

Thanatin coding sequence was codon optimized for efficient expression in *Pichia pastoris* with GeneArt software (Thermo Fisher Scientific, Waltham, MA, USA). The corresponding sequence flanked by spacer sequences for homology recombination cloning was obtained by PCR using P11 and P12 primers (Supplementary Table S2). The HiFi DNA assembly kit (New England Biolabs, UK) was used to subclone the PCR product into the expression vector pGAP4_MCS digested with BsaI restriction endonuclease. The resulting expression vector pGAP4_rThan was identified by PCR and verified by sequencing.

### 4.3. Yeast Transformation, rThan Production and Purification

The yeast strain *Pichia pastoris* GS115 was transformed by electroporation according to protocol [50] with the pGAP4_rThan plasmid linearized at the AvrII site. Transformed cells were selected on RDB plates (1.34% (*w*/*v*) yeast nitrogen base, 0.00004% (*w*/*v*) biotin, 2% (*w*/*v*) glucose, 1M sorbitol, 1.8% (*w*/*v*) agar, 0.005% of L-glutamic acid, L-methionine, L-lysine, L-leucine, and L-isoleucine) supplemented with 100 µg/mL of ampicillin and kanamycin. The overnight culture of the selected yeast colony was used to inoculate 100 mL of buffered YPD medium (1% yeast extract, 2% peptone, 2% glucose, 100 mM potassium phosphate pH 6.0) with final optical density A_600_ = 1. Recombinant yeasts were cultured at 30 °C in 750 mL shake-flasks at 180 rpm for 48 h. Culture medium was clarified by centrifugation at 5000 rpm for 5 min and filtered through a 0.22 um membrane. Filtered culture media was applied to a SP Sepharose (GE Healthcare, Chicago, IL, USA) 1 mL column pre-equilibrated with 20 mM ammonium acetate pH 5.8 (buffer A) and eluted with a linear gradient of buffer B (20 mM ammonium acetate pH 5.8, 1 M sodium chloride) from 0 to 100%. Elution fractions were analyzed by Tricine-sodium dodecyl sulfate-polyacrylamide gel electrophoresis (Tricine-SDS-PAGE). rThan concentration was verified after Coomassie Brilliant Blue gel staining. Purified rThan was dialyzed overnight in 20 mM Tris-HCl buffer pH 7.4 and stored at −20 °C. The final yield was estimated as 20 mg per 1 L of culture media.

### 4.4. Agar Overlay Assay

For agar overlay assay, *P. pastoris* clones were grown on YPD-agar plates (1% yeast extract, 2% peptone, 2% glucose, 100 mM potassium phosphate pH 6.0, 1.8% agar) for 2 days at 30 °C. Soft agar (8 g/L tryptone, 2.5 g/L NaCl, 5 g/L yeast extract, 0.5% agar) was melted, cooled to 42 °C, and inoculated with *E. coli* Δ*tolC* to a final concentration of approximately 10^6^ CFU/mL. *P. pastoris* colonies were then overlaid with inoculated soft agar and incubated at 37 °C overnight.

### 4.5. Cocultivation with Target Bacteria

For the cocultivation assay, the *E. coli* Δ*tolC* strain producing sfGFP as a detection marker was constructed according to previous work [51]. Cocultivation of rThan yeast with target bacteria was provided according to the protocol described previously [35]. Briefly, *E. coli* Δ*tolC* sfGFP and *P. pastoris* rThan overnight cultures were diluted with 2YT medium (16 g/L tryptone, 10 g/L yeast extract, 5 g/L NaCl) in 96-well culture plates using two-fold serial dilutions starting with 4 × 10^8^ and 10^8^ CFU/mL, respectively, and incubated at 30 °C overnight with vigorous shaking. sfGFP fluorescence was measured with a Varioskan Flash multimode reader (Thermo Fisher Scientific, USA). One hundred percent target growth corresponded to a fluorescence level at wells that contain target *E. coli* Δ*tolC* sfGFP only. Background level corresponded to a fluorescence of wells that did not contain target *E. coli* Δ*tolC* sfGFP.

### 4.6. Peptide Chemical Synthesis

#### 4.6.1. Materials

N,N-Dimethylformamide (DMF), dichloromethane (DCM), and methyl tert-butyl ether (MTBE) for peptide synthesis were purchased from Vecton (St. Petersburg, Russia). Acetonitrile for high-pressure liquid chromatography (HPLC) was purchased from Honeywell Specialty Chemicals GmbH (Seelze, Germany). Fmoc-protected amino acids (Fmoc-Gly-OH, Fmoc-Arg(Pbf)-OH, Fmoc-Asn(Trt)-OH, Fmoc-Cys(Trt)-OH, Fmoc-Gln(Trt)-OH, Fmoc-Ile-OH, Fmoc-Lys(Boc)-OH, Fmoc-Met-OH, Fmoc-Ser(tBu)-OH, Fmoc-Thr(tBu)-OH, Fmoc-Tyr(tBu)-OH, Fmoc-Val-OH, and Fmoc-Pro-OH), 2-chlorotrityl chloride resin (100–200 mesh), and O-(Benzotriazol-1-yl)-N,N,N′,N′-tetramethyluronium hexafluorophosphate (HBTU) for solid-phase synthesis were obtained from Iris Biotech GmbH (Marktredwitz, Germany). Trifluoroacetic acid (TFA), triisopropylsilane (TIS), and N-methylmorpholine (NMM) were obtained from Sigma-Aldrich (Darmstadt, Germany). All other chemicals were purchased from Sigma-Aldrich (Darmstadt, Germany).

#### 4.6.2. Peptide Synthesis

The linear precursor of sThanatin was synthesized by the solid-phase peptide synthesis method on a scale of 0.1 mmol. Then, 250 mg of 2-chlorotrityl chloride resin (100–200 mesh, Iris Biotech) was swelled in dichloromethane (DCM) for 30 min in a polypropylene vessel [52,53]. The first amino acid was directly coupled to the resin using 2% N-methylmorpholine (NMM) in DCM, with shaking for 1 h on a Heidolph Multi Reax shaker. The resin was then capped by adding methanol directly into the reaction vessel. Subsequent amino acids were coupled (without preactivation) using 0.4 mmol of Fmoc-protected amino acid, 0.4 mmol of HBTU coupling agent, and 0.8 mmol NMM in dimethylformamide (DMF), with shaking for 1 h [54]. Between amino acid couplings, the Fmoc-protecting group was removed via two 10 min agitations with 20% 4-methylpiperidine in DMF [55]. Removal of the final Fmoc-protecting group completed the peptide synthesis. The peptide was cleaved from the resin via a 2 h reaction with a cleavage cocktail consisting of 95:2.5:2.5 TFA:TIS:water [56]. Following cleavage, the resin was washed with TFA and DCM, and the volume of the cleavage solution was reduced by evaporation with nitrogen. The peptide solution was then transferred into cold methyl tert-butyl ether (MTBE) to precipitate the peptide. Centrifugation at 4000 rpm for 5 min pelleted the peptide. The peptide was dried, then dissolved in a minimal amount of 5% acetic acid and lyophilized.

#### 4.6.3. Disulfide Bond Formation in Thanatin

The crude-reduced thanatin precursor (50 mg, 0.02 mmol, HPLC purity 52.3%) was introduced into a 100 mL round-bottom flask, and a mixture of water and acetonitrile (1:1, 50 mL) was added and then stirred for approximately 15 min. Initially, the measured pH, being 2.5, was adjusted to 6.5 by adding NH_4_OH (10%) dropwise. After the mixture was mechanically stirred for 22 h at 350 rpm, at room temperature, the reaction was quenched by adding TFA, adjusting the pH to 2.5. The reaction mixture was then lyophilized without further evaporation [57]. The crude thanatin was obtained with 46% HPLC purity. The molecular mass of the oxidized peptide was certified using mass spectrometry MALDI-TOF data (*m*/*z*): [M + H]^+^ 2433.34 found; 2432.27 calculated.

#### 4.6.4. Peptide Purification

Preparative HPLC runs were performed on a Symmetry C18 column (19 × 300 mm, 5 μm, Waters, Eschborn, Germany), using linear gradients water–acetonitrile (with 0.1% TFA) 5–60% for 60 min with a flow rate of 8 mL/min (Figure 3). Fractions with more than 95% HPLC homogeneity and with the expected molecular mass of the peptide were combined, lyophilized, and used in subsequent experiments.

### 4.7. Evaluation of Antimicrobial Activity

The minimal inhibitory concentrations (MIC) were estimated with a microwell broth dilution assay. In brief, tested bacteria were diluted in 2YT media (10 g/L yeast extract, 16 g/L tryptone, 5 g/L NaCl) to a final concentration of approximately 1 × 10^6^ CFU/mL in a 96 microwell plate and the rThan sample was added to generate two-fold serial dilutions. Serial dilutions of ciprofloxacin were used as a control for antimicrobial activity. Plates were incubated at 37 °C with shaking at 450 rpm. MIC was determined as the lowest concentration of peptide that inhibits the growth of tested bacteria after 16 h of incubation at 37 °C. MICs were determined in triplicate and analyzed by *t*-test and non-parametric (Mann–Whitney) tests using Prism 7 (GraphPad) software.

### 4.8. Evaluation of Cytotoxicity

The cytotoxicity of thanatin toward human cells was determined by the MTT assay [58]. Briefly, HEK 293T cells were cultured in DMEM (Gibco, Waltham, MA, USA) supplemented with 10% fetal bovine serum (Gibco, USA) and GlutaMAX (Gibco, USA). Cells were seeded in a 96-well plate at a concentration of 1× 10^4^ cells/well and incubated in a 5% CO_2_ incubator at 37 °C. After 16 h, cells were exposed to various concentrations (1–256 μM) of thanatin for 72 h. After thanatin treatment, MTT (Sigma-Aldrich, USA) solution in DPBS (5 mg/mL) was added to a final concentration of 0.45 mg/mL, followed by a 2 h incubation at 37 °C. Formazan crystals were dissolved by the addition of an equal volume of solubilization solution (40% (*v*/*v*) dimethylformamide (Sigma-Aldrich, USA), 2% (*v*/*v*) glacial acetic acid (Sigma-Aldrich, USA), and 16% (*w*/*v*) sodium dodecyl sulfate (Sigma-Aldrich, USA). Finally, the absorbance was measured at 570 nm using a Varioskan Flash multimode reader (Thermo Fisher Scientific, USA). MICs were determined in triplicate and analyzed by *t*-test using Prism 7 (GraphPad) software.

### 4.9. Liquid Chromatography and Mass Spectrometry

Samples were loaded into a trap column 50 × 0.1 mm, packed with Inertsil ODS3 3 mm (GL Sciences, Tokyo, Japan) sorbent (Dr. Maisch, Ammerbuch, Germany), in the loading buffer (2% ACN, 98% H_2_O, 0.1% TFA) at 4 mL/min flow, and separated at RT in a home-packed [59] fused-silica column 300 × 0.1 mm packed with Reprosil PUR C18AQ 1.9 (Dr. Maisch, Germany) into an emitter prepared with P2000 Laser Puller (Sutter, Atlanta, GA, USA). Reverse-phase chromatography was performed with an Ultimate 3000 Nano LC System (Thermo Fisher Scientific, USA), which was coupled to the Q Exactive Plus Orbitrap mass spectrometer (Thermo Fisher Scientific, USA) via a nano electrospray source (Thermo Fisher Scientific, USA). Peptides were loaded in a loading solution (98% 0.1% (*v*/*v*) formic acid, 2% (*v*/*v*) acetonitrile) and eluted with a linear gradient: 3–6% B for 3 min, 6–25% B for 30 min, 25–40% B for 25 min, 40–60% B for 4 min, 60% B for 3 min, 60–99% B for 0.1 min, 99% B for 10 min, and 99–2% B for 0.1 min at a flow rate of 500 nl/min. Buffer A was 5% acetonitrile and 0.1% formic acid and buffer B was 80% acetonitrile and 0.1% formic acid. MS1 parameters were as follows: 70K resolution, 350–1600 scan range, max injection time—35 msec, and AGC target—3 × 10^6^. Ions were isolated with 1.4 *m*/*z* window, preferred peptide match, and isotope exclusion. Dynamic exclusion was set to 30 s. MS2 fragmentation was carried out in HCD mode at 17.5 K resolution with HCD collision energy 30%, max injection time—80 ms, and AGC target—1 × 10^5^. Other settings: charge exclusion—unassigned, 1, >7.

## 5. Conclusions

Here, a self-maintaining source of antimicrobial activity was engineered by the constitutive expression of AMP thanatin in the yeast *P. pastoris*. A simple purification scheme was adopted for the high-scale production of recombinant thanatin (rThan). rThan was equivalent to the chemically synthesized thanatin, mediating antibacterial activity on a panel of bacteria, including the MDR pathogen *Klebsiella pneumoniae* 0980. The model target bacterium *E. coli* Δ*tolC* was efficiently inhibited by bioengineered yeasts in a cocultivation assay. These results will facilitate research in the field of directed creation of biocontrol agents. Further improvement in the antimicrobial activity of thanatin may be achieved by increasing the permeability of the outer membrane with respective antimicrobials such as pore-forming AMP or antibiotics. Overall, the achieved results support a growing trend in the consideration of AMP-based antibiotics as a part of antimicrobial strategies. We believe that yeast bioengineering and AMP development will provide novel prospects for the construction of living biocontrol agents and the implementation of alternative strategies to counteract antibiotic resistance.

## Data Availability

The data presented in this study are contained within the article and Supplementary Materials.

## Supplementary Materials

The following supporting information can be downloaded at https://www.mdpi.com/article/10.3390/antibiotics12121719/s1: Figure S1: MS spectrum of rThan obtained by electrospray LC-MS; Table S1: List of plasmids used in this study; Table S2: List of oligonucleotides used in this study.
