# Supplementary material for: Bioengineering the Antimicrobial Activity of Yeast by Recombinant Thanatin Production
Source: Antibiotics (Basel). 2023 Dec 12;12(12):1719. doi: 10.3390/antibiotics12121719 (PMC10741026; doi:10.3390/antibiotics12121719)
Supplement: Supplementary file 1 [file antibiotics-12-01719-s001.zip › antibiotics-2767368-supplementary.pdf]

Supplementary information for “Bioengineering the Antimicro-bial Activity of Yeast by Recombinant Thanatin Production”

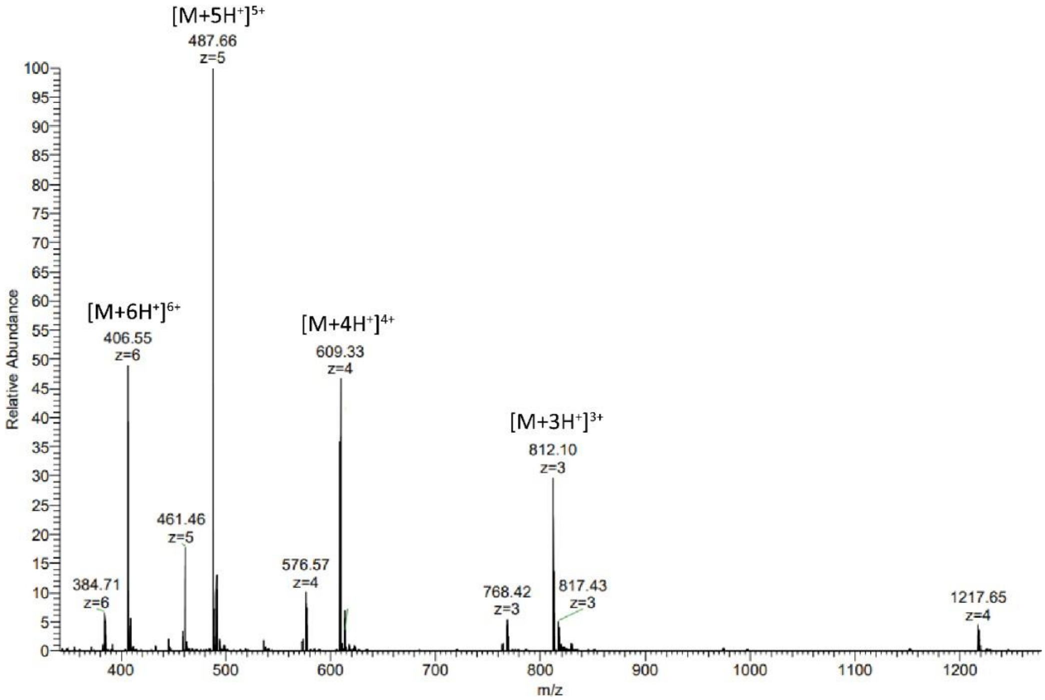

Figure S1. MS spectrum of rThan obtained by electrospray LC-MS.

Table S1. List of plasmids used in this study.

Plasmid Name	Plasmid Source
pYTK002	MoClo YTK [1]
pPTK002	MoClo PTK [2]
pPTK006	MoClo PTK [2]
pPTK017	MoClo PTK [2]
pPTK019	MoClo PTK [2]
pYTK072	MoClo YTK [1]
pYTK095	MoClo YTK [1]
pLvL1_GAP_1	This study
pLvL1_GAP_MCS	This study
pPIC9K	Invivogen
pGAP4_MCS	This study
pGAP4_rThan	This study

Table S2. List of oligonucleotides used in this study.

**P1**	**TCAAGAGGATGTCAGAATGCC**
P2	TCTTTTCTCCAGAGATACCCCTTC
P3	AGGGGTATCTCTGGAGAAAAGAGGAGACCGAGGATCCAGATCTCATATGCCT
P4	GGCATTCTGACATCCTCTTGACTCATGAGACCCTTAAGGCATATGAGATCTGGATC CTCG
P5	TGAGTTTGTAGCCTTAGACATGAC
P6	GTTTATGCTTTTTCTGAAACCGCAAAGTTGG
P7	CCAACTTTGCGGTTTCAGAAAAAGCATAAAC
P8	CGGCCGCTCGGCGCTCTCCCTTATGCGACTC
P9	AAGGGAGAGCGCCGAGCGGCCGCGATTATCA
P10	CATGTCTAAGGCTACAAACTCATGAGACCCTTAAGGCA
P11	AGGGGTATCTCTGGAGAAAAGAGGTTCCAAGAAGCCAGTTCCAATCATCTACTGCAACAGAC-GTACCGGTAA
P12	CATGTCTAAGGCTACAAACTCACATTCTCTGGCACTTACCGGTACGTCTGTT

References

1. Lee, M.E.; DeLoache, W.C.; Cervantes, B.; Dueber, J.E. A Highly Characterized Yeast Toolkit for Modular, Multipart Assembly. *ACS Synth. Biol.* **2015**, *4*, 975–986. https://doi.org/10.1021/sb500366v.
2. Obst, U.; Lu, T.K.; Sieber, V. A Modular Toolkit for Generating *Pichia Pastoris* Secretion Libraries. *ACS Synth. Biol.* **2017**, *6*, 1016–1025. https://doi.org/10.1021/acssynbio.6b00337.
